# Exploring the impact of religiosity and spirituality on depressive symptoms in homeless people

**DOI:** 10.1192/j.eurpsy.2024.679

**Published:** 2024-08-27

**Authors:** P. H. F. Camargo, J. V. G. N. de Moraes, L. M. Vitorino

**Affiliations:** ^1^Medicine, Faculty of Medicine of Itajubá; ^2^Medicine, Faculty of Medicine of Itajubá, Itajubá, Brazil

## Abstract

**Introduction:**

Depression is a major concern among homeless individuals. Studies link religiosity and spirituality (RS) with lesser depressive symptoms, but evidence is scarce among the homeless.

**Objectives:**

This study aims to assess the association between RS and depressive symptoms in homeless individuals in Brazil.

**Methods:**

This cross-sectional study involved 456 homeless individuals in São Paulo, Brazil. It received approval from the Ethics and Research Committee of the Faculty of Medicine of Itajubá, Brazil. We used adjusted linear regression models to analyze the association between RS and participants’ depressive symptoms. Depressive symptoms were assessed with the Patient Health Questionnaire-9 (PHQ-9). We used the P-DUREL to measure religiosity, FACIT-Sp12 for spirituality, and the Brief-RCOPE scale for religious-spiritual coping strategies.

**Results:**

Out of 482 invited participants, 456 (94.6%) completed all questionaries, mostly males (75%) with an average age of 44.53 (SD 12.62) years. About 49.6% had depressive symptoms (PHQ-9 ≥10 points). After controlling for sociodemographic and health variables, factors such as temple/church attendance (≥ 3 times per month), increased religiousness (both organizational and intrinsic), positive religious/spiritual coping, and peace, faith and meaning were inversely related to depressive symptoms. Conversely, dysfunctional use of RS, such as in negative spiritual-religious coping strategies, correlated with heightened depressive symptoms.

**Conclusions:**

High depressive symptom prevalence was found among Brazilian homeless individuals. Functional use of RS was negatively linked to depressive symptoms, while dysfunctional RS, like negative spiritual-religious coping strategies, correlated with higher depressive symptoms. These findings can aid healthcare professionals, particularly psychologists and psychiatrists, in addressing RS in the homeless population.

**Disclosure of Interest:**

None Declared

